# Outborn Neonates in Nairobi’s Public Hospitals: Mortality Burden and Associated Risk Factors in a Retrospective Cohort Study

**DOI:** 10.12688/wellcomeopenres.25241.1

**Published:** 2026-04-13

**Authors:** John Wainaina, John Gachohi, Jalemba Aluvaala

**Affiliations:** 1 School of Public Health, Jomo Kenyatta University of Agriculture and Technology, Nairobi, Nairobi County, 00200, Kenya; 2 Graduate School, Kenya Medical Research Institute, Nairobi, Nairobi County, 00100, Kenya; 3 Global Health Kenya, Washington State University, Nairobi, Kenya, 00100, Kenya; 4 Washington State University, Paul G, Allen School of Global Health, Pullman, WA, 99164, USA; 5 Department of Paediatrics and Child Health, University of Nairobi, Nairobi, Nairobi County, 00100, Kenya; 6 Health Services and Implementation Cluster, KEMRI-Wellcome Trust Research Programme Nairobi, Nairobi, Nairobi County, 00100, Kenya

**Keywords:** neonatal mortality, outborn neonates, neonatal referral, Population Attributable Fraction (PAF), referral distance

## Abstract

**Background:**

Neonatal mortality remains a significant challenge in Sub-Saharan Africa. Outborn neonates - those born outside the admitting hospital - are particularly vulnerable, yet their outcomes remain under-characterized in many low-resource urban settings. This study examines the burden, characteristics, and mortality risk among outborn neonates admitted to public neonatal units in Nairobi, Kenya.

**Methods:**

We conducted a retrospective cohort study across four high-volume public hospitals in Nairobi County, Kenya. A stratified random sample of 400 outborn neonates admitted between January and December 2023 was reviewed using medical records. To estimate outborn prevalence and the Population Attributable Fraction (PAF) for mortality, we analyzed registry data from 9,021 neonatal admissions with complete outcome and place-of-birth information. Referral distances were calculated using geocoded coordinates of referring and receiving facilities. Multivariable logistic regression identified factors independently associated with inpatient neonatal mortality.

**Results:**

Outborn neonates accounted for 17% of neonatal admissions, with a mortality rate of 25.6%, double that of inborn (12.5%; RR=2.05, p<0.001). The PAF was 15.0% (95% CI: 12.3-17.7%), indicating that one in seven neonatal deaths could be attributed to outborn status. Key factors were significantly associated with increased odds of mortality including; extremely low birth weight (<1000g; AOR=7.8, 95% CI: 2.6, 23), very low birth weight (1000-1499g; AOR= 4.7 95% CI:2.3, 9.7), hypothermia (AOR=2.2, 95% CI: 1.1, 4.5), respiratory distress (AOR=2.1, 95% CI: 1.2, 3.8), and referral distance >12.1 km (AOR=2.6, 95% CI: 1.1, 6.6). Most deaths (68%) occurred within three days of admission.

**Conclusion:**

Outborn neonates in Nairobi’s public hospitals face substantially higher early mortality, primarily driven by low birth weight, hypothermia, respiratory distress, and longer referral distances. Interventions targeting improved stabilization, timely referral, and early neonatal care are urgently needed. These findings provide actionable evidence to inform policies aimed at reducing neonatal mortality and advancing progress toward SDG 3.2.

## Plain Language summary

Neonatal death - deaths that occur within the first 28 days of life - remains a major health challenge in many low-resource settings, including urban areas of Kenya. Babies born outside hospitals (“outborn” neonates) are often at higher risk of dying soon after birth, but there is limited information about their outcomes in busy public hospitals. In this study, we looked at nearly 9,000 newborn admissions across four public hospitals in Nairobi and conducted a detailed review of 400 outborn babies admitted during 2023. We found that outborn neonates made up 17% of admissions but accounted for a much higher proportion of deaths, with one in four dying in the hospital, double the risk compared with babies born in the hospital. Factors such as very low birth weight, difficulty breathing, low body temperature, and longer travel distances to the hospital were linked to higher death rates. Most deaths occurred within the first three days after admission. These findings highlight the urgent need for timely transfer, proper stabilization, and early care for babies born outside hospitals to reduce preventable deaths and improve newborn survival.

## Background

Neonatal mortality remains a significant global health challenge, particularly in Sub-Saharan Africa (SSA). In 2023, the neonatal mortality rate was 26 deaths per 1,000 live births in SSA. ^1^ However, achieving the Sustainable Development Goal (SDG) 3.2 target of reducing neonatal mortality to 12 per 1,000 live births by 2030 demands accelerated implementation of effective, evidence-based interventions in high-burden regions like SSA. ^2^ These interventions must prioritize access to essential life-saving measures, including skilled delivery during birth and high-quality postnatal care. ^2^

Among these vulnerable populations, outborn neonates – referrals in, and other categories of babies born outside the admitting hospital, such as at home, during transit, or at other healthcare facilities - represent a unique group with heightened risks of morbidity and mortality. ^3^ Though less studied, the burden of outborn neonates varies widely, ranging from 9% to 49% of neonatal intensive care unit (NICU) admissions in different settings, with a disproportionately higher prevalence among neonates with low birth weight (1000–2500 g). ^3, 4^ Outborn neonates are often admitted with complications such as respiratory distress syndrome, infections, and feeding difficulties, which require specialized care at higher-level neonatal units. ^4^

In Kenya, neonatal mortality remains a pressing issue, with rates higher than the global average - 21 deaths per 1,000 live births in 2022. ^5^ Nairobi County, in particular, faces considerable challenges, with a neonatal mortality rate of 20 deaths per 1,000 live births. ^5^ Four public hospitals in Nairobi County handle approximately 71% of 24/7 inpatient neonatal cases, with 15% of these admissions being outborn neonates. ^6, 7^ Despite their clinical vulnerability, the outcomes and care trajectories of these outborn neonates remain poorly characterized.

This study aimed to generate evidence on outborn neonatal care in these high-volume public hospitals by estimating their prevalence among neonatal admissions, quantifying the mortality burden attributable to outborn status, and identifying clinical and demographic predictors of mortality. In addition, it explored how referral sources and distances travelled were associated with outcomes.

## Methods

### Study setting and design

This retrospective cohort study was conducted in the Neonatal Units (NBUs) of four public hospitals located in Nairobi County, Kenya. Kenyatta National Hospital (KNH) is a national tertiary referral and teaching hospital providing Level III neonatal care, which includes advanced respiratory support, intensive monitoring, and management of critically ill or very low birth weight neonates. ^8^ The remaining three hospitals – Mama Lucy Kibaki Hospital (MLKH), Mbagathi County Hospital (MCH), and Pumwani Maternity Hospital (PMH) are county-level public hospitals offering Level II neonatal care, such as oxygen therapy, thermal care, assisted feeding, and treatment for common neonatal conditions. ^9, 10^ MLKH and MCH are general hospitals providing a range of medical services including neonatal care, while PMH is a specialized maternity hospital. All four serve as referral centers for newborns from lower-level facilities within Nairobi County and surrounding regions.

### Study population and sampling

This study reviewed the medical records of outborn neonates (i.e., neonates born outside the admitting hospital) admitted to the four NBUs between January 1 and December 31, 2023. Admission registers were used to generate a sampling frame of all outborn admissions. A simple random sampling approach was used to select 400 records-100 per hospital-to ensure adequate statistical power for subgroup analyses and facilitate balanced inter-hospital comparisons. This strategy minimized selection bias and maintained representativeness within each facility. The sample size was calculated based on an expected outborn neonatal mortality prevalence of 26%, a 95% confidence level, a 5% margin of error, and a design effect of 1.2 to account for clustering across the four hospitals. An additional 20% was added to compensate for potential missing or incomplete records, resulting in a final sample of 400 records.

In addition to the sampled outborn data, aggregate summaries of total neonatal admissions, inborn and outborn admissions, and corresponding mortality counts during the same 12-month period were extracted directly from the official NBU admission registers (MOH 373) maintained at each hospital. These data were used to estimate the prevalence of outborns among all admissions and to calculate the population attributable fraction (PAF) of neonatal deaths due to outborn status.

### Data collection

Trained Health Records and Information Officers abstracted data from the standard Ministry of Health neonatal admission and discharge forms using a REDCap electronic tool hosted at the KEMRI-Wellcome Trust Research Programme, Nairobi (Harris et al., 2009) (Michuki et al., 2018). Extracted variables included sociodemographic details, referral information, clinical status on admission (e.g., temperature, respiratory signs), comorbidities, interventions, and outcomes.

### Geocoding and distance measurement

Geospatial analysis was limited to a subset of outborn neonates who were referred from identifiable health facilities, as accurate geolocation was not feasible for other outborns (e.g., home births or births in undocumented facilities). Facility names for referring hospitals were abstracted from patient records and standardized. Geographic coordinates (latitude and longitude) for both referring and receiving hospitals were obtained from the Kenya Master Facility List (KMFL). ^11^ Straight-line distances (in kilometers) between referring and receiving facilities were computed using the Haversine formula, which calculates great-circle distances between two points on the Earth’s surface ^12^:

d=2r∗arcsin(sqrt(sin^2(Δφ/2)+cos(φ1)∗cos(φ2)∗sin^2(Δλ/2)))

*Where:*

*d = distance between two points (in km)*

*r = Earth's radius (6,371 km)*

*ϕ1,ϕ2 = latitudes (in radians)*

*Δϕ = ϕ2−ϕ1*

*Δλ = λ2−λ1*

*λ1,λ2 = longitudes (in radians)*

This approach accounts for the Earth’s curvature and provides a reasonable approximation of physical travel distance, which was used as a proxy for referral distance in the analysis. ^12^ Distances were categorized into quartiles for regression analysis to allow for flexible modeling of potential non-linear effects and to reduce the influence of extreme outliers.

### Population Attributable Fraction (PAF) calculation

To estimate the proportion of neonatal deaths attributable to being outborn, the Population Attributable Fraction (PAF) was calculated using the following formula ^13^:

PAF=Pe(RR−1)/Pe(RR−1)+1

*Where:*

*p𝑒 is the proportion of the population exposed (i.e., proportion of outborn neonates among all admissions),*

*RR is the relative risk of mortality for outborn versus inborn neonates.*

### Statistical analysis

Descriptive statistics were used to summarize baseline characteristics and outcome frequencies. To examine factors associated with mortality, mixed-effects logistic regression modeling was applied. The hospital of admission was included as a random intercept to account for clustering.

Modeling was performed using an imputed dataset, with Multiple Imputation by Chained Equations (MICE) applied to covariates with ≤20% missingness. Variables included in the final model were distance category, facility level of referring hospital, birth weight category, thermal condition at admission, and presence of respiratory distress. Results were reported as adjusted odds ratios (AORs) with 95% confidence intervals. Statistical significance was set at p < 0.05. All analyses were conducted using R statistical software (v4.3.2).

### Ethical considerations

This study used retrospectively collected data from routine medical records; individual informed consent was not required, and all data were fully deidentified using unique study identifiers. Ethical approval for this study was obtained from the Kenyatta National Hospital - University of Nairobi Ethics and Scientific Review Committee (Approval No. P402/04/2024).

## Results

### Demographic characteristics and clinical conditions on admission

Most neonates were male (60%, n=240), and more than half (57%, n=227) had birth weights between 2500-4000g. Extremely low birth weight (<1000g) and very low birth weight (1000–1499g) accounted for 5.3% (n=21) and 14% (n=56) of admissions, respectively. Among the 354 neonates with gestational age data, 61% (n=218) were term. Median age on admission was 13 hours (IQR: 4–24), with KNH having the longest delays. Most mothers (89%, n=287) were aged 18-35 years, and maternal death was rare (1%) Table 1.

**Table 1.** Demographic, clinical characteristics and outcomes of outborn neonates.

Characteristic	Overall N = 400 *^1^*	KNH N = 100 *^1^*	MLKH N = 100 *^1^*	MCH N = 100 *^1^*	PMH N = 100 *^1^*
**Baby's sex**					
*Female*	160 (40%)	39 (39%)	40 (40%)	43 (43%)	38 (38%)
*Male*	240 (60%)	61 (61%)	60 (60%)	57 (57%)	62 (62%)
**Birth weight**					
*2500-4000g*	227 (57%)	46 (46%)	60 (60%)	63 (63%)	58 (58%)
*<1000g*	21 (5.3%)	9 (9.0%)	4 (4.0%)	5 (5.0%)	3 (3.0%)
*1000-1499g*	56 (14%)	18 (18%)	11 (11%)	13 (13%)	14 (14%)
*1500-1999g*	49 (12%)	9 (9.0%)	14 (14%)	11 (11%)	15 (15%)
*2000-2499g*	40 (10%)	14 (14%)	9 (9.0%)	7 (7.0%)	10 (10%)
*>4000g*	7 (1.8%)	4 (4.0%)	2 (2.0%)	1 (1.0%)	0 (0%)
**Gestational age**					
*<28 Weeks*	18 (5.1%)	8 (9.0%)	6 (6.7%)	2 (2.3%)	2 (2.2%)
*≥37 Weeks*	218 (61%)	48 (54%)	58 (65%)	49 (56%)	63 (70%)
*28-32 Weeks*	61 (17%)	18 (20%)	10 (11%)	19 (22%)	14 (16%)
*33-36 Weeks*	59 (17%)	15 (17%)	15 (17%)	18 (20%)	11 (12%)
*Missing*	44	11	11	12	10
**Age on admission (hours)**	13 (4, 24)	24 (6, 144)	7 (3, 24)	24 (7, 72)	5 (3, 21)
*Missing*	76	27	28	11	10
**Maternal age**					
*<18 Years*	8 (2.5%)	1 (1.3%)	2 (2.7%)	2 (2.5%)	3 (3.3%)
*>35 Years*	28 (8.7%)	6 (7.5%)	6 (8.2%)	8 (10%)	8 (8.8%)
*18-35 Years*	287 (89%)	73 (91%)	65 (89%)	69 (87%)	80 (88%)
*Missing*	77	20	27	21	9
**Maternal hypertension**	12 (3.5%)	6 (7.6%)	0 (0%)	6 (6.7%)	0 (0%)
*Missing*	53	21	14	10	8
**Temperature Status**					
*Hyperthermia*	36 (9.7%)	5 (5.6%)	2 (2.0%)	22 (24%)	7 (7.6%)
*Normothermia*	182 (49%)	35 (39%)	64 (65%)	43 (47%)	40 (43%)
*Cold Stress*	89 (24%)	17 (19%)	28 (29%)	16 (18%)	28 (30%)
*Moderate Hypothermia*	61 (16%)	31 (35%)	4 (4.1%)	9 (9.9%)	17 (18%)
*Severe Hypothermia*	2 (0.5%)	1 (1.1%)	0 (0%)	1 (1.1%)	0 (0%)
*Missing*	30	11	2	9	8
**Oxygen Saturation**					
*<90%*	68 (19%)	18 (21%)	16 (17%)	12 (14%)	22 (26%)
*≥90%*	285 (81%)	69 (79%)	79 (83%)	75 (86%)	62 (74%)
*Missing*	47	13	5	13	16
**APGAR Score**					
*7-10*	238 (70%)	48 (59%)	70 (77%)	66 (80%)	54 (65%)
*0-3*	6 (1.8%)	1 (1.2%)	2 (2.2%)	1 (1.2%)	2 (2.4%)
*4-6*	94 (28%)	32 (40%)	19 (21%)	16 (19%)	27 (33%)
*Missing*	62	19	9	17	17
**Resuscitated at Birth**	86 (30%)	36 (40%)	23 (29%)	17 (22%)	10 (22%)
*Missing*	109	10	22	23	54
**Respiratory Distress**	95 (24%)	35 (35%)	22 (22%)	25 (25%)	13 (13%)
*Missing*	0	0	0	0	0
**Jaundice**	25 (7.0%)	10 (10%)	0 (0%)	13 (14%)	2 (2.3%)
*Missing*	43	0	21	8	14
**LBW/Prematurity**	140 (35%)	46 (46%)	31 (31%)	31 (31%)	32 (32%)
**Infections**	125 (31%)	39 (39%)	12 (12%)	60 (60%)	14 (14%)
**Neonatal Jaundice**	32 (8.0%)	4 (4.0%)	1 (1.0%)	24 (24%)	3 (3.0%)
**Respiratory Distress Syndrome (RDS)**	159 (40%)	33 (33%)	55 (55%)	37 (37%)	34 (34%)
**Intrapartum Complications**	137 (34%)	34 (34%)	36 (36%)	21 (21%)	46 (46%)
**Congenital Deformities**	4 (1.0%)	3 (3.0%)	1 (1.0%)	0 (0%)	0 (0%)
**Other Diagnoses**	26 (6.5%)	9 (9.0%)	7 (7.0%)	2 (2.0%)	8 (8.0%)
**Outcome**					
*Alive*	284 (71.2%)	61 (61.0%)	73 (73.0%)	73 (73.0%)	77 (78.8%)
*Dead*	106 (26.5%)	38 (38.0%)	24 (24.0%)	22 (22.0%)	22 (22.2%)
*Referred*	9 (2.3%)	1 (1.0%)	3 (3.0%)	5 (5.0%)	0 (0.0%)
*Missing*	1	0	0	0	1
**Length of Stay (All)**					
*>7 Days*	190 (48%)	57 (58%)	48 (48%)	55 (55%)	30 (30%)
*≤3 Days*	113 (28%)	22 (22%)	27 (27%)	23 (23%)	41 (41%)
*4-7 Days*	95 (24%)	19 (19%)	25 (25%)	22 (22%)	29 (29%)
*Missing*	2	2	0	0	0
**Length of Stay (Survivors)**					
*>7 Days*	169 (58%)	47 (78%)	43 (57%)	54 (69%)	25 (32%)
*≤3 Days*	41 (14%)	2 (3.3%)	9 (12%)	5 (6.4%)	25 (32%)
*4-7 Days*	81 (28%)	11 (18%)	24 (32%)	19 (24%)	27 (35%)
**Length of Stay (Deaths)**					
*>7 Days*	21 (20%)	10 (26%)	5 (21%)	1 (4.5%)	5 (23%)
*≤3 Days*	72 (68%)	20 (53%)	18 (75%)	18 (82%)	16 (73%)
*4-7 Days*	13 (12%)	8 (21%)	1 (4.2%)	3 (14%)	1 (4.5%)

KNH – Kenyatta National Hospital |MLKH – Mama Lucy Kibaki Hospital |MCH – Mbagathi County Hospital|PMH – Pumwani Maternity Hospital

^1^ n (%); Median (Q1, Q3)

Of the 370 neonates with documented admission temperature, 40.5% were hypothermic (24% cold stress, 16% moderate, 0.5% severe). SpO _2_ levels were <90% in 19% of neonates, with the highest rate at PMH (26%). APGAR scores were available for 338 neonates: 70% scored 7-10, 28% scored 4-6, and 2% scored 0-3. Resuscitation at birth was reported in 30% of cases. Respiratory distress was noted in 24%, jaundice in 7% Table 1.

Common diagnoses included respiratory distress syndrome (40%), low birth weight/prematurity (35%), and neonatal infections (31%). Intrapartum complications were reported in 34% of neonates, especially at PMH (46%). Jaundice (8%) and congenital anomalies (1%) were less frequent Table 1.

Among the 400 outborn neonates, 71% (n = 284) survived to discharge, while 27% (n = 106) died during hospitalization. A small proportion (2%, n = 9) were referred elsewhere, and outcome data was missing for one neonate. Mortality varied by hospital: 38% at KNH, 24% at MLKH, and 22% each at MCH and PMH. Most deaths (68%) occurred within the first 3 days, particularly at MCH (82%) and PMH (73%). Only 20% of deaths occurred after 7 days of hospitalization. Among survivors, 58% stayed longer than 7 days, particularly at KNH (78%). Short stays (≤3 days) were common at PMH (32%) but rare at KNH (3.3%) Table 1.

### Prevalence and Referral Patterns

Per the annual admissions aggregate data from the newborn unit registers, 10421 neonates were admitted to the newborn units (NBUs) of four public hospitals in Nairobi County between January and December 2023. Among these, 1515 (17%) were outborn - delivered outside the receiving facility and referred for inpatient care - while 7512 (83%) were inborn. Birth location data were missing for 1394 neonates and were excluded from facility-level estimates. The proportion of outborns varied across hospitals: 22% at Mbagathi County Hospital (MCH) (446/2,067), 18% at Mama Lucy Kibaki Hospital (MLKH) (438/2,480), 16% at Kenyatta National Hospital (KNH) (283/1,785), and 13% at Pumwani Maternity Hospital (PMH) (348/4,089) Table 2. These findings highlight a substantial referral burden on urban NBUs.

**Table 2.** Prevalence of outborn admissions among annual admissions.

Characteristic	Overall N = 10,421	Kenyatta National Hospital N = 1,785	Mama Lucy Kibaki Hospital N = 2,480	Mbagathi County Hospital N = 2,067	Pumwani Maternity Hospital N = 4,089
**Place of birth**					
*Inborn*	7,512 (83%)	1,502 (84%)	2,042 (82%)	1,621 (78%)	2,347 (87%)
*Outborn*	1,515 (17%)	283 (16%)	438 (18%)	446 (22%)	348 (13%)
*Missing*	1,394	0	0	0	1,394
**Outcome**					
*Alive*	8,935 (86%)	1,390 (78%)	2,086 (84%)	1,787 (86%)	3,672 (90%)
*Died*	1,474 (14%)	395 (22%)	394 (16%)	280 (14%)	405 (9.9%)
*Missing*	12	0	0	0	12

Among the 400 outborn neonatal records reviewed (100 per hospital), most (91.5%) were referred from other health facilities, while 8.5% were born before arrival (BBA).

Referral origin varied widely by site: KNH primarily received neonates from secondary-level hospitals (81%), while MLKH admitted over half from primary care facilities (53%). PMH and MCH had more diverse referral sources. Analysis by facility level showed that outborn neonates were commonly referred from level 3 (33%) and level 4 (35%) hospitals, with smaller proportions from level 2 (21%) and level 5 (11%) facilities. PMH and MLKH received higher proportions of referrals from lower-level facilities. The distance from referring to receiving facilities was variable.

A quarter of neonates traveled ≤3.4 km, another 25% between 3.5–7.3 km, and the remaining half from 7.4–12.1 km (26%) or more than 12.1 km (24%). Distance varied by site: 46% of neonates at KNH traveled >12.1 km, compared to only 14% at PMH. Conversely, nearly half of MLKH outborns came from ≤3.4 km Table 3.

**Table 3.** Referral patterns and outcomes among the outborn.

Characteristic	Overall N = 400 *^1^*	KNH N = 100 *^1^*	MLKH N = 100 *^1^*	MCH N = 100 *^1^*	PMH N = 100 *^1^*
**Place of birth**					
*Born before arrival (BBA)*	34 (8.5%)	4 (4.0%)	5 (5.0%)	13 (13.0%)	12 (12.0%)
*Referred In*	366 (91.5%)	96 (96.0%)	95 (95.5%)	87 (87.0%)	88 (88.0%)
**outcome**					
*Alive*	284 (71.2%)	61 (61.0%)	73 (73.0%)	73 (73.0%)	77 (78.8%)
*Dead*	106 (26.5%)	38 (38.0%)	24 (24.0%)	22 (22.0%)	22 (22.2%)
*Referred*	9 (2.3%)	1 (1.0%)	3 (3.0%)	5 (5.0%)	0 (0.0%)
*Missing*	1	0	0	0	1
**Facility type**					
*Other/Private Facility*	75 (24%)	13 (16%)	9 (15%)	28 (32%)	25 (29%)
*Primary Care Facility*	84 (27%)	3 (3.6%)	32 (53%)	13 (15%)	36 (42%)
*Secondary Hospital*	156 (50%)	67 (81%)	19 (32%)	46 (53%)	24 (28%)
*Missing*	85	17	40	13	15
**Facility level**					
*2*	67 (21%)	4 (4.8%)	16 (27%)	17 (20%)	30 (35%)
*3*	103 (33%)	12 (14%)	25 (42%)	29 (33%)	37 (44%)
*4*	111 (35%)	36 (43%)	18 (30%)	39 (45%)	18 (21%)
*5*	34 (11%)	31 (37%)	1 (1.7%)	2 (2.3%)	0 (0%)
*Missing*	85	17	40	13	15
**Distance to hospital**					
*Q1 (≤3.4km)*	75 (25%)	13 (16%)	27 (45%)	10 (12%)	25 (34%)
*Q2 (3.5–7.3km)*	74 (25%)	9 (11%)	24 (40%)	16 (19%)	25 (34%)
*Q3 (7.4–12.1km)*	78 (26%)	21 (27%)	3 (5.0%)	40 (47%)	14 (19%)
*Q4 (>12.1km)*	71 (24%)	36 (46%)	6 (10%)	19 (22%)	10 (14%)
*Missing*	102	21	40	15	26

^1^ n (%)|BBA - neonates delivered outside a health facility (typically at home or en route) who are subsequently brought to a hospital for care, often without formal referral documentation.

### Population Attributable Mortality (PAF) from outborn status

Among 9,021 neonates with complete outcome and place-of-birth data in the newborn unit registers, the mortality rate was 25.6% (95% CI: 23.5%–27.9%) among outborns and 12.5% (95% CI: 11.8%–13.3%) among inborns, yielding a relative risk (RR) of 2.05. This indicates that outborn neonates were twice as likely to die during hospitalization compared to their inborn counterparts (χ ^2^ = 171.6, p < 0.001). The population attributable fraction (PAF) for outborn status was 15.0% (95% CI: 12.3%–17.7%), suggesting that under an assumption of causality, nearly one in seven neonatal deaths could be attributed to being born outside the receiving facility.

### Factors associated with neonatal mortality

Multivariable logistic regression identified several significant factors associated with increased odds of inpatient neonatal mortality among outborns. Compared to neonates weighing ≥2500g, those with birth weight <1000g had 7.8 times the odds of death (95% CI: 2.6-23.0), and those weighing 1000-1499g had 4.7 times the odds (95% CI: 2.3-9.7). Hypothermia on admission was associated with higher mortality (OR = 2.2; 95% CI: 1.1-4.5; p = 0.026), as was respiratory distress (OR = 2.1; 95% CI: 1.2-3.8; p = 0.010). Sex, APGAR scores, oxygen saturation, place of birth, maternal factors, delivery mode, and clinical diagnosis of infections were not significantly associated with mortality Table 4.

**Table 4.** Factors associated with mortality among outborn neonates.

Characteristic	Crude OR (95% CI)	p-value	Adjusted OR ^1^ (95% CI)	p-value
**Birth weight**				
≥2500g	—		—	
<1000g	11 (4.1, 32)	**<0.001**	7.8 (2.6, 23)	**<0.001**
1000-1499g	5.0 (2.7, 9.3)	**<0.001**	4.7 (2.3, 9.7)	**<0.001**
1500-1999g	0.97 (0.41, 2.1)	>0.9	0.89 (0.38, 2.1)	0.8
2000-2499g	1.1 (0.44, 2.4)	0.9	0.80 (0.32, 2.0)	0.6
**APGAR score category**				
7-10	—		—	
4-6	1.1 (0.76, 1.6)	0.5	0.97 (0.53, 1.8)	>0.9
0-3	0.24 (0.05, 0.93)	**0.047**	2.4 (0.54, 11)	0.3
**Sex of child**				
Female	—		—	
Male	1.1 (0.82, 1.6)	0.5	1.2 (0.83, 1.8)	0.3
**Place of birth**				
Referred In	—		—	
BBA	0.84 (0.34, 1.8)	0.7	0.92 (0.34, 2.5)	0.9
**Maternal age**				
18-35 Years	—		—	
<18 Years	1.9 (0.39, 8.1)	0.4	1.8 (0.37, 8.5)	0.5
>35 Years	1.5 (0.63, 3.4)	0.3	1.9 (0.76, 4.6)	0.2
**Maternal complications**				
No	—		—	
Yes	0.92 (0.29, 2.4)	0.9	0.71 (0.20, 2.6)	0.6
**Mode of delivery**				
Spontaneous vaginal (SVD)	—		—	
Caesarean section (C/S)	0.80 (0.38, 1.6)	0.6	1.0 (0.45, 2.3)	>0.9
Others	0.77 (0.11, 3.3)	0.8	0.47 (0.07, 3.1)	0.4
**Thermal status**				
Normothermia	—		—	
Cold Stress	0.52 (0.25, 1.0)	0.066	0.54 (0.27, 1.1)	0.083
Hypothermia	4.4 (2.4, 8.2)	**<0.001**	2.2 (1.1, 4.5)	**0.026**
Hyperthermia	0.67 (0.24, 1.6)	0.4	0.78 (0.28, 2.2)	0.6
**Oxygen saturation**				
≥90%	—		—	
<90%	2.1 (1.2, 3.7)	**0.008**	1.6 (0.90, 3.0)	0.11
**Respiratory distress**				
No	—		—	
Yes	2.6 (1.6, 4.3)	**<0.001**	2.1 (1.2, 3.8)	**0.010**
**Clinical infections**				
No	—		—	
Yes	0.93 (0.57, 1.5)	0.8	0.90 (0.50, 1.6)	0.7
**Distance travelled**				
Q1 (≤3.4km)	—		—	
Q2 (3.5–7.3km)	0.87 (0.40, 1.9)	0.7	0.81 (0.29, 2.2)	0.7
Q3 (7.4–12.1km)	1.5 (0.74, 3.1)	0.3	2.2 (0.88, 5.5)	0.10
Q4 (>12.1km)	1.6 (0.79, 3.4)	0.2	2.6 (1.1, 6.6)	**0.041**
**Facility level**				
High-level	—		—	
Low-level	0.69 (0.34, 1.4)	0.3	0.77 (0.31, 1.9)	0.6
Mid-level	0.85 (0.43, 1.7)	0.6	0.76 (0.34, 1.7)	0.5
Tertiary-level	2.5 (1.1, 6.0)	**0.034**	0.46 (0.17, 1.3)	0.14

^1^ OR = Odds Ratio, CI = Confidence Interval

Neonates referred from >12.1 km had significantly increased odds of death compared to those from ≤3.4 km (OR = 0.39; 95% CI: 0.15-0.95; p = 0.041) sources. A similar trend was seen for distances of 7.4-12.1 km (OR = 0.46; p = 0.10), although did not reach significance thresholds. Referring facility level was not significantly associated with mortality.

## Discussion

This study provides a detailed examination of the clinical characteristics, outcomes, and predictors of mortality among outborn neonates admitted to four public hospitals in Nairobi County. Our findings reveal that outborn neonates comprised nearly one in six admissions and faced a significantly higher risk of mortality compared to their inborn counterparts, with a population attributable fraction of 15%. These results underscore the persistent vulnerability of neonates referred after birth and highlight opportunities for strengthening referral systems and early neonatal care.

These findings are broadly consistent with prior work by Wainaina et al. (2023), which described referral patterns and outcomes across three public first-referral hospitals in Nairobi. In both studies, referred neonates had a high burden of prematurity and low birth weight, with mortality among referred-in neonates reaching approximately 26%. ^7^ However, our study expands on previous insights by including a national tertiary facility, estimating the population-level contribution of outborn deaths, and integrating referral distance analysis using geospatial methods. Together, these additions offer a more comprehensive understanding of how interfacility referral processes influence neonatal survival, and they reinforce the urgency of improving triage, stabilization, and transport practices across levels of care.

The overall mortality rate among outborn neonates in this study was 26%, twice that observed among inborn neonates. This finding aligns with evidence from other low-resource settings. For instance, Rakholia et al. (2014) reported mortality rates of 25% among outborns compared to 15% among inborns in a tertiary facility in India, underscoring the heightened vulnerability of referred neonates. ^14^ This elevated risk likely reflects a combination of delayed recognition of illness, suboptimal resuscitation or stabilization at the birth site, and gaps in referral processes - including delayed transport and inadequate thermal or respiratory support during transit previously identified to predictors of neonatal mortality among outborn neonates. ^15– 17^ As previously noted by Dol et al. (2023), early neonatal mortality - especially within the first seven days - is commonly associated with birth asphyxia, complications of prematurity, congenital anomalies, and severe infections. ^18^ In the present study, two-thirds of deaths among outborn neonates occurred within the first three days of admission, highlighting a critical window of vulnerability.

A substantial proportion of outborn neonates were hypothermic at admission (40.5%), and hypothermia independently predicted mortality in this cohort. This prevalence is more than twice that previously reported among hospitalized neonates in Kenya (18% across 21 hospitals), where estimates were limited to inborn neonates admitted on the day of birth. ^19^ These findings highlight the persistent and preventable burden of hypothermia as a contributor to neonatal morbidity and mortality. ^19, 20^ This problem is often exacerbated by prolonged transit times and inadequate thermal care during referral. ^21^ The median admission age of 13 hours further reflects delays in access to inpatient care. These findings align with regional evidence that early referral and stabilization are critical to neonatal survival. ^22^

Low birth weight, particularly 1000-1499g, was one of the strongest predictor of mortality (aOR: 4.7, 95% CI: 2.3, 9.7, p < 0.001) in the regression model. This finding reinforces the central role of prematurity and or low birth weight in neonatal outcomes and echoes various estimates that neonatal deaths are more associated with low birth weight and or preterm neonates. ^23– 25^ Although only a minority of outborn neonates were extremely low birth weight, their disproportionately high risk of death underscores the need for specialized, high-acuity care and early identification at birth.

Neonates referred from more than 12 km away had markedly lower odds of survival, even after adjusting for key clinical risk factors. This likely reflects the combined impact of delayed access to care, deterioration during transit, and inadequate pre-referral stabilization. Evidence from Ethiopia similarly underscores the effect of distance: for every 10 km increase in travel to a health facility, the odds of neonatal mortality rose by 1.33% (95% CI: 1.06%-1.67%). ^26^ Greater distance has also been linked to lower utilization of antenatal, delivery, and postnatal services, highlighting systemic access barriers. ^26^ Notably, the level of the referring facility was not independently associated with survival, suggesting that the timeliness and continuity of care, may be more critical than the formal classification of the referring institution influencing neonatal outcomes.

### Strengths and limitations

This study leverages multisite data to analyze outborn neonatal outcomes in urban Kenya, key for generalizability. However, it is limited by missing data on unmeasured confounding (e.g., severity of illness at birth, antenatal care data, transport/referral methods, care during transit), which may influence some associations. Nevertheless, the use of multivariable regression and consistent patterns across facilities enhance the robustness of the findings.

## Conclusion

Outborn neonates admitted to Nairobi’s public NBUs face high early mortality, largely driven by low birth weight and or prematurity, hypothermia, respiratory distress, and referral distance. Strengthening perinatal and referral care, across all levels of care, is essential to improving survival. These findings should inform policy and practice aimed at reducing neonatal deaths under the SDG framework.

## Data Availability

The data used in this study were derived from routinely collected patient records obtained from participating public hospitals and the Ministry of Health. These data are not fully publicly available because they contain sensitive individual-level health information and the authors are not the primary data owners. The relevant ethical approval and data governance review explicitly permitted the use of these data for approved secondary analyses but restricted unrestricted public release to protect patient confidentiality and comply with national data protection requirements. Access to the deidentified dataset and associated geocoded data is therefore subject to controlled access. Requests for data access can be submitted to the KEMRI-Wellcome Trust Research Programme (KWTRP) Data Governance Committee ( dgc@kemri-wellcome.org). Access is granted to reviewers and researchers following review of a data request application, in line with KWTRP’s data sharing policy and governance framework. Full details of the data access process, eligibility criteria, and conditions for reuse-including the data request form-are available at: https://dataverse.harvard.edu/dataverse/kwtrp. Subject to approval, the deidentified dataset and associated geocoded data are hosted in Harvard Dataverse and can be accessed via the following DOI: http://doi.org/10.7910/DVN/L6XJID. ^27^ Data dictionaries, codebooks, analysis scripts, and all materials required to reproduce the results are openly available in the same repository. Harvard Dataverse. Outborns Outcome in 4 Nairobi County Hospitals. http://doi.org/10.7910/DVN/L6XJID. ^27^ This project contains the following extended data:
•
Outborn_neonatal_dataset_deidentified.csv • (Deidentified individual-level dataset of outborn neonatal admissions, including demographic, clinical, referral, and outcome variables.) •
Geocoded_referral_data.tab • (Geographic coordinates of referring and receiving health facilities used to calculate referral distances.) • Data_codebook.tab • (Detailed definitions, variable labels, and coding schemes for all variables included in the analytic dataset.) •
Data_dictionary_and_readme.tab • (Overview of the dataset structure, inclusion criteria, and guidance on data use and interpretation.) •
Data_manipulation_script.R • (R script used for data cleaning, variable derivation, and preparation of analytic datasets.) •
Descriptive_analysis_script.R • (R script used to generate descriptive statistics and summary tables.) •
Confidence_intervals_script.R • (R script used to estimate confidence intervals and Population Attributable Fraction measures.) Data is available under the terms of the CC BY 4.0license.
