# Supplementary material for: Outborn Neonates in Nairobi's Public Hospitals: Mortality Burden and Risk Factors
Source: Wellcome Open Res. 2026 Aug 4;11:217. Originally published 2026 Apr 13. [Version 2] doi: 10.12688/wellcomeopenres.25241.2 (PMC13560558; doi:10.12688/wellcomeopenres.25241.2)
Supplement: Peer review history

# Reviewer report: Reviewer response for version 2

**DOI:** 10.21956/wellcomeopenres.30077.r163287

**Published:** 2026-08-06

**Authors:** Hassib Narchi

**Affiliations:** 1 United Arab Emirates University, Abu Dhabi, United Arab Emirates

I am happy with the modifications made.

Is the work clearly and accurately presented and does it cite the current literature?

Yes

If applicable, is the statistical analysis and its interpretation appropriate?

Partly

Are all the source data underlying the results available to ensure full reproducibility?

No source data required

Is the study design appropriate and is the work technically sound?

Partly

Are the conclusions drawn adequately supported by the results?

Partly

Are sufficient details of methods and analysis provided to allow replication by others?

Yes

Reviewer Expertise:

NA

I confirm that I have read this submission and believe that I have an appropriate level of expertise to confirm that it is of an acceptable scientific standard.

# Reviewer report: Reviewer response for version 2

**DOI:** 10.21956/wellcomeopenres.30077.r163288

**Published:** 2026-08-05

**Authors:** Alexander G Stevenson

**Affiliations:** 1 African Neonatal Network, Kigali, Zimbabwe

Thank you- my points have been answered

Is the work clearly and accurately presented and does it cite the current literature?

Yes

If applicable, is the statistical analysis and its interpretation appropriate?

Yes

Are all the source data underlying the results available to ensure full reproducibility?

Yes

Is the study design appropriate and is the work technically sound?

Yes

Are the conclusions drawn adequately supported by the results?

Yes

Are sufficient details of methods and analysis provided to allow replication by others?

Yes

Reviewer Expertise:

neonatology, neonatal mortailty analysis.

I confirm that I have read this submission and believe that I have an appropriate level of expertise to confirm that it is of an acceptable scientific standard.

# Reviewer report: Reviewer response for version 1

**DOI:** 10.21956/wellcomeopenres.27818.r153418

**Published:** 2026-05-12

**Authors:** Alexander G Stevenson

**Affiliations:** 1 African Neonatal Network, Kigali, Zimbabwe

This an important paper exploring increased mortality amongst babies born outside the treating facility, in keeping with other studies. And the call to action for the paper is that perhaps 1 in 7 babies deaths could be avoided by better interventions targeting stabilisation, timely referral and early neonatal care.

 Although the strengths and limitations section says the use of multivariable regression enhances the robustness of the findings, I may need a bit more explicit explanation of what was done in this logistic regression. From my reading it seems to be that in addition to being outborn, being small, or cold, or coming from far were additional independent risk factors for death. My question is whether any attempt was made to compare the outborn babies with similar inborn babies. It is likely that outborn babies are likely to be smaller and sicker than inborn, and less likely to be exposed to antenatal steroids. How did the outcomes of babies 1000-1500g compare inborn and outborn?

 Another possible limitation of this study is that we are only seeing the babies who arrived at the referral hospital, whereas a more " goldstandard" denominator may be the babies who were referred from the clinics and other hospitals. Some may never arrive at the referral hospital because they die, or maybe the parents just take them home.

 Finally, I was intrigued by the significant and consistent sex difference amongst referred babies- any theory as to why this may be?

Is the work clearly and accurately presented and does it cite the current literature?

Yes

If applicable, is the statistical analysis and its interpretation appropriate?

Yes

Are all the source data underlying the results available to ensure full reproducibility?

Yes

Is the study design appropriate and is the work technically sound?

Yes

Are the conclusions drawn adequately supported by the results?

Yes

Are sufficient details of methods and analysis provided to allow replication by others?

Yes

Reviewer Expertise:

neonatology, neonatal mortailty analysis.

I confirm that I have read this submission and believe that I have an appropriate level of expertise to confirm that it is of an acceptable scientific standard, however I have significant reservations, as outlined above.

# Response

**Published:** 2026-07-14

**Authors:** John Wainaina

**Affiliations:** KEMRI Wellcome Trust Research Programme, Kenya

*We thank Dr. Stevenson for his supportive review and for raising several substantive analytical and interpretive questions. We address each below.*

 Although the strengths and limitations section says the use of multivariable regression enhances the robustness of the findings, I may need a bit more explicit explanation of what was done in this logistic regression. From my reading it seems to be that in addition to being outborn, being small, or cold, or coming from far were additional independent risk factors for death. My question is whether any attempt was made to compare the outborn babies with similar inborn babies. It is likely that outborn babies are likely to be smaller and sicker than inborn, and less likely to be exposed to antenatal steroids. How did the outcomes of babies 1000-1500g compare inborn and outborn?

*A direct matched comparison of outborn vs. inborn neonates by birth weight was outside this study's scope - our detailed abstraction was collected only for the outborn cohort, and the aggregate inborn registry data lack individual-level covariates.* *For context, however, Irimu et al.'s Clinical Information Network study of 41,657 inborn neonates across 16 Kenyan public hospitals ( https://gh.bmj.com/content/6/5/e004475) - including all four hospitals in the present study - reported substantial birth-weight-stratified mortality among inborn, with threefold variation across hospitals in the 1000–1499g and 1500–1999g categories. This suggests low birth weight carries markedly elevated mortality risk even among inborn neonates in the same hospital network, consistent with birth weight acting as a dominant risk factor independent of birth location. We have cited this in the Discussion as relevant context, while being clear it is not a matched or adjusted comparison and cannot tell us how much of our crude RR=2.05 reflects birthweight confounding versus an independent outborn effect - a question we continue to flag as requiring prospective, matched-cohort data in future work.*  

 Another possible limitation of this study is that we are only seeing the babies who arrived at the referral hospital, whereas a more " goldstandard" denominator may be the babies who were referred from the clinics and other hospitals. Some may never arrive at the referral hospital because they die, or maybe the parents just take them home. *This is an important and correct point about referral/survivorship bias that we did not adequately address. Neonates who die in transit, at the referring facility, or whose parents decline transfer never enter our sampling frame (which is drawn from admission registers at the four receiving hospitals). This means our mortality estimates for outborn neonates are conditional on having survived to reach and be admitted at a referral hospital - the "true" population-level mortality burden associated with referral delay/failure is understated, not overstated, by our figures. We have added this explicitly to Limitations, framed as: our results likely represent a conservative estimate of the total mortality burden linked to outborn status and referral, since pre-arrival deaths and non-completed referrals are excluded by design.*

 Finally, I was intrigued by the significant and consistent sex difference amongst referred babies- any theory as to why this may be?

*Looking back at Table 1, males were 60% of outborn admissions overall (consistent 57–62% across all four hospitals) - but sex was not significantly associated with mortality in the regression – Table 4 (OR 1.1 crude, 1.2 adjusted, both non-significant). So the "significant and consistent" pattern our reviewer could be referring to is the male-predominance in referral/admission itself, not a sex-mortality association. We wish to clarify this distinction in the reply, since conflating the two would misrepresent our own findings.* *On the admission-sex-skew itself: this is a well-documented pattern in neonatal epidemiology - male neonates have consistently higher rates of prematurity-related complications, respiratory distress, congenital malformations, etc. across many settings, plausibly linked to slower fetal lung maturation and higher susceptibility to hypoxic and respiratory morbidity in male infants relative to female infants of the same gestational age. We note this as a plausible biological explanation and here are some links you could explore: https://pmc.ncbi.nlm.nih.gov/articles/PMC4675056/ https://www.sciencedirect.com/science/article/abs/pii/S0301211514006265* *We did not test these directly and it is offered as context, not a finding of this study.*

# Reviewer report: Reviewer response for version 1

**DOI:** 10.21956/wellcomeopenres.27818.r153419

**Published:** 2026-04-30

**Authors:** Olufunke Bolaji

**Affiliations:** 1 Paediatrics, Federal Teaching Hospital Ido-Ekiti (Ringgold ID: 605037), Ido Ekiti, Ekiti, Nigeria

Thank you for the opportunity to review this manuscript which is a retrospective cohort study on the mortality burden and associated risk factors of outborn neonates in Nairobi's public hospitals.

 The paper is generally well written providing a background of neonatal mortality globally, in the region and in Kenya. It builds a justification for evaluating the mortality in outborn babies by providing evidence on the proportion of outborn babies seen in Nairobi county and how complications drives the mortality seen in them.

 The study design and statistical tools are appropriate for the study aims. The results show the disproportionately higher mortality in outborn babies and a population attributable factor of 15%. The study also identified the key factors associated with increased risk of mortality especially hypothermia and low birth weight. The discussion addressed some of the critical findings.

 To improve the quality of the manuscript, I would suggest

 1. A more concise title e.g. Mortality and associated risk factors of outborn neonates in Nairobi's public hospitals.

 2. Improve clarity of sentence in the background: "among these vulnerable populations”: sentence not quite explicit on which population is being referred to.

 3. Arrange categories of GA sequentially under Table 1

 4. Provide clarity on how the diagnosis of Respiratory distress syndrome was made because the total numbers of LBW/Prematurity (the population in which RDS would occur) in MLKH for example is 31; yet RDS occurred in 55 babies; similarly for PMH, 34 babies with RDS when only 32 were LBW/Preterm babies

 5. Arrange categories of length of days sequentially

 6. Categories should read < 3 days for length of stay for survivors and deaths and ALL since this was what was referred to in the prose.

 7. In the discussion, a more recent reference than Rakholia et al. (2014) would emphasize the point being discussed.

 Overall, the manuscript contributes significantly to evidence on newborn mortality from low resource settings.

Is the work clearly and accurately presented and does it cite the current literature?

Yes

If applicable, is the statistical analysis and its interpretation appropriate?

Yes

Are all the source data underlying the results available to ensure full reproducibility?

Yes

Is the study design appropriate and is the work technically sound?

Yes

Are the conclusions drawn adequately supported by the results?

Yes

Are sufficient details of methods and analysis provided to allow replication by others?

Yes

Reviewer Expertise:

Neonatology, preterm births, respiratory distress syndrome

I confirm that I have read this submission and believe that I have an appropriate level of expertise to confirm that it is of an acceptable scientific standard, however I have significant reservations, as outlined above.

# Response

**Published:** 2026-07-14

**Authors:** John Wainaina

**Affiliations:** KEMRI Wellcome Trust Research Programme, Kenya

We thank Dr. Bolaji for her positive assessment and constructive suggestions. We address each point below.

 1. **More concise title.**

*We appreciate the suggestion. However, we'd like to retain "Burden," since the population-level mortality burden (prevalence of outborn admissions, attributable fraction) is one of the paper's two central findings, alongside the individual-level risk factor analysis - dropping it would understate that contribution. We propose instead: "Outborn Neonates in Nairobi's Public Hospitals: Mortality Burden and Risk Factors," which shortens the title while preserving both core results.*

 2. **Clarity on "among these vulnerable populations" in the Background.**

*Agreed - the antecedent is unclear in the original sentence: We have revised the sentence to:* *"Neonates in Sub-Saharan Africa remain broadly at elevated risk of death, but outborn neonates – referrals in, and other categories of babies born outside the admitting hospital, such as at home, during transit, or at other healthcare facilities - represent a unique sub-group with heightened risks of morbidity and mortality."*

 3. **Arrange GA categories sequentially in Table 1.**

*Agreed - we have reordered across in Tables 1 through 4.*

 4. **RDS diagnosis count inconsistency vs. LBW/Prematurity denominator.**

*This is a good catch and concern. At MLKH, LBW/Prematurity = 31 but RDS = 55; at PMH, 32 vs. 34 - counts that would be implausible if RDS occurred only as a complication of prematurity/LBW. In practice, "respiratory distress syndrome" was recorded as a clinical working diagnosis at admission (based on presentation) rather than a confirmed surfactant-deficiency diagnosis, and diagnosis fields were not mutually exclusive or nested - a neonate could carry an RDS diagnosis independent of how LBW/Prematurity was coded. We have clarified this in Table 1 to help readers appreciate that fact.*

 5. **Arrange length-of-stay categories sequentially.**

*Agreed - we have reordered across all levels as appropriate across all tables.*

 6. **Categories should read < 3 days for length of stay for survivors and deaths and ALL since this was what was referred to in the prose.**

*We have streamlined the tables and prose to read as intended.*  

 7. **More recent reference than Rakholia et al. (2014).**

*We have added a recent study from Uganda (2023) to strengthen that discussion.*

# Reviewer report: Reviewer response for version 1

**DOI:** 10.21956/wellcomeopenres.27818.r153412

**Published:** 2026-04-27

**Authors:** Hassib Narchi

**Affiliations:** 1 United Arab Emirates University, Abu Dhabi, United Arab Emirates

This research article examines the high mortality rates and risk factors for "outborn" neonates—infants born outside the facility where they are eventually treated—across four public hospitals in Nairobi, Kenya. They conclude with the need to improve stabilisation practices and more efficient referral systems to reduce preventable infant deaths and meet international health goals.

 The authors are to be commended for addressing this important public health care problem in Kenya. The resulting recommendations, when backed by robust analysis, should provide an actionable plan for implementation and improving the outcomes of outborn neonates.

 I am taking the liberty to share with the authors my suggestions to improve and strengthen this work.

 METHODS

 The source of the expected outborn mortality prevalence of 26% used to calculate the sample size should be mentioned (previous studies, hospital audits, etc,). 

  The Geocoding method and analysis to calculate the distances involved in the transfer is very interesting but not accurate. it was applied only when the sources of referrals were hospitals. Furthermore, it calculated a straight line between the referring and the admitting centre which is not always correct. It does not take into consideration the traffic conditions on these roads (congestions, rush hours) nor the state of these roads (maintained in good conditions, narrow or not, not always on a flat terrain)not the time of the day for each transfer (less congestion at night, etc). It would have been more realistic and relevant if the time taken for each transfer was used instead of the geospatial distance, although the authors acknowledge its difficulty. These issues may impact on the results of the study and hence, the recommendations made.

 With many missing variable data (between 15 and 27%), the multiple inputation (MICE) method which was used might not be correct as it is only adequate and reliable if less than 29% of the data are missing. This issue would certainly impact on the results and their interpretation.

RESULTS

 The calculation of the Population Attributable Mortality for outborn neonates should explicitly describe if the relative risk (RR) used in the calculation was the crude or the adjusted RR, as the result and its interpretation are likely to be different.

 In the paragraph Factors associated with mortality, the authors should clarify if the OR with 95% conficence intervals refers to the raw OR or the adjusted OR. The neonates referred over a distance > 12 km had an adjusted OR of death of 2.6 when compared to a shorter distance and not 0.39 as reported, this would change completely the interpretation of that paragraph.

DISCUSSION

 LBW as a predictor of mortality should not be restricted to 1000-1499 g, but also include < 1000 g

TABLE 1. All the variables should be logically ordered by their values to facilitate reading.

 In all the rows mentioning the number of missing data, the percentage of missing data should be added.

 The row Resuscitation at birth is very vague: ventilatory support, ?chest compression? medications? 

 One row mentions respiratory distress and another one is labelled distress syndrome. Probably the authors meant surfactant deficiency for the latter and other cuases for the former (transient tachypnea, meconium aspiration, pneumonia, etc). This should be clarified.

 Another column reports LBW and prematurity together, although they do not mean exactly the same thing. This could be confusing.

TABLE 2

 The total on inborn + outborn neonates per year is 9026 and not 9021 as stated in the text.

TABLE 4

 All the variables should be logically ordered by their values to facilitate reading.

OVERALL

 In addition, the authors should address all the discrepancies and contradictions between the text and the tables.

CONCLUSIONS

 I hope that the authors find these suggestions helpful and improve the manuscript which should make strong contribution to neonatal health issues in Sub-Sharan Africa and help achieve the Sustainable Development Goal (SDG) of reducing neonatal mortality.

Is the work clearly and accurately presented and does it cite the current literature?

Yes

If applicable, is the statistical analysis and its interpretation appropriate?

Partly

Are all the source data underlying the results available to ensure full reproducibility?

No source data required

Is the study design appropriate and is the work technically sound?

Partly

Are the conclusions drawn adequately supported by the results?

Partly

Are sufficient details of methods and analysis provided to allow replication by others?

Yes

Reviewer Expertise:

Paediatrics and perinatology

I confirm that I have read this submission and believe that I have an appropriate level of expertise to confirm that it is of an acceptable scientific standard, however I have significant reservations, as outlined above.

# Response

**Published:** 2026-07-14

**Authors:** John Wainaina

**Affiliations:** KEMRI Wellcome Trust Research Programme, Kenya

**REVIEWER 1: DR. NARCHI**

*We thank Dr. Narchi for a thorough and constructive review. We have addressed each point below and indicated the corresponding manuscript revision.*

***METHODS***

 The source of the expected outborn mortality prevalence of 26% used to calculate the sample size should be mentioned (previous studies, hospital audits, etc,). 

*We agree this needs an explicit citation. This estimate was drawn from Wainaina et al. (2023) (ref. 7), which reported ~26% mortality among referred-in neonates across three first-referral hospitals in Nairobi – largely similar population base extended in the present study. We have added this citation directly to the Methods sentence.*

 The Geocoding method and analysis to calculate the distances involved in the transfer is very interesting but not accurate. it was applied only when the sources of referrals were hospitals. Furthermore, it calculated a straight line between the referring and the admitting centre which is not always correct. It does not take into consideration the traffic conditions on these roads (congestions, rush hours) nor the state of these roads (maintained in good conditions, narrow or not, not always on a flat terrain)not the time of the day for each transfer (less congestion at night, etc). It would have been more realistic and relevant if the time taken for each transfer was used instead of the geospatial distance, although the authors acknowledge its difficulty. These issues may impact on the results of the study and hence, the recommendations made.

*We agree this is a genuine limitation and did not intend to imply the Haversine distance approximates actual transit experience. We have expanded the Strengths and Limitations paragraph to explicitly state that straight-line distance does not capture road quality, congestion, time-of-day effects, or actual travel time, and that this may bias the distance–mortality association toward or away from the null in ways we cannot determine with available data. We have noted that time-stamped transfer data would be a valuable addition for future work, as the reviewer suggests. *  

 With many missing variable data (between 15 and 27%), the multiple inputation (MICE) method which was used might not be correct as it is only adequate and reliable if less than 29% of the data are missing. This issue would certainly impact on the results and their interpretation.

*Imputation was restricted to the covariates included in the final regression model, and only those with ≤20% missingness - this is stated in the Methods ("MICE was applied to covariates with ≤20% missingness"). The 15–27% range reflects missingness across all variables abstracted in Table 1, not the subset modeled; several higher-missingness variables (e.g., resuscitation at birth, 27% missing) were not imputed or included in the regression at all.*

*We have revised the Methods sentence to make this distinction explicit.* *On the general reliability of MICE at this level of missingness: we don't think a fixed percentage cutoff is the right standard, as there is no consensus threshold in the missing-data literature (e.g., White, Royston & Wood, 2011, Stat Med https://doi.org/10.1002/sim.4067 , show MICE performing well beyond 20–30% missingness under Missing at Random (MAR) with adequately specified models). However, we agree that results would be more unreliable with increasing missingness.*  

***RESULTS***

 The calculation of the Population Attributable Mortality for outborn neonates should explicitly describe if the relative risk (RR) used in the calculation was the crude or the adjusted RR, as the result and its interpretation are likely to be different.

*The RR of 2.05 used in the PAF calculation was the crude RR, derived directly from the raw mortality rates (25.6% vs 12.5%) in the full registry dataset (which lacks individual-level covariates needed for adjustment). We will state this explicitly in the Results and note it as a limitation - the PAF estimate assumes the crude RR approximates the causal effect of outborn status, which may be confounded by unmeasured factors (birth weight, gestational age, etc., which are disproportionately more common among outborn admissions).*

 In the paragraph Factors associated with mortality, the authors should clarify if the OR with 95% conficence intervals refers to the raw OR or the adjusted OR. The neonates referred over a distance > 12 km had an adjusted OR of death of 2.6 when compared to a shorter distance and not 0.39 as reported, this would change completely the interpretation of that paragraph.

*Thank you for catching a real error. We have corrected the Results text to read: "Neonates referred from >12.1 km had significantly increased odds of death compared to those from ≤3.4 km (aOR = 2.6; 95% CI: 1.1–6.6; p = 0.041)... A similar trend was seen for distances of 7.4–12.1 km (aOR = 2.2; 95% CI: 0.88–5.5; p = 0.10)." We have also re-checked all other aOR values transcribed into prose throughout Results and Discussion against Table 4 to rule out similar transcription errors elsewhere.*  

***DISCUSSION***

 LBW as a predictor of mortality should not be restricted to 1000-1499 g, but also include < 1000 g

*We agree and this was an oversight. Extremely low birth weight (<1000g) carried an even stronger association (aOR 7.8, 95% CI 2.6–23.0) than the 1000–1499g category. We have revised the paragraph to lead with the <1000g finding and discuss both categories together as evidence of a birth-weight gradient in mortality risk.*  

TABLE 1. All the variables should be logically ordered by their values to facilitate reading.

 In all the rows mentioning the number of missing data, the percentage of missing data should be added.

 The row Resuscitation at birth is very vague: ventilatory support, ?chest compression? medications? 

 One row mentions respiratory distress and another one is labelled distress syndrome. Probably the authors meant surfactant deficiency for the latter and other cuases for the former (transient tachypnea, meconium aspiration, pneumonia, etc). This should be clarified.

 Another column reports LBW and prematurity together, although they do not mean exactly the same thing. This could be confusing.

*We have reordered all categorical variables into ascending/logical order. We have also added missing-data percentages alongside each missing-data row count.* *"Resuscitation at birth" has been clarified as bag-mask ventilation, based on what the abstraction tool captured.* *We have updated "Respiratory distress" to “respiratory distress signs” (clinical signs of inner chest wall indrawing and or severe grunting during admission examination)* *We have clarified that "LBW/Prematurity" reflects a single combined diagnosis category as recorded in the original medical records and abstraction tool - i.e., clinicians entered "low birth weight/prematurity" as one diagnostic label at the point of care. We have added a table footnote stating this explicitly, so readers don't mistake it for a derived variable and understand why its counts don't nest cleanly with the standalone birth weight or gestational age rows.*    

TABLE 2

 The total on inborn + outborn neonates per year is 9026 and not 9021 as stated in the text.

*Thank you for pointing this out, however, upon rechecking total against the source registry data, we note that (10,421 − 1,394 missing = 9,027 - univariate datapoint) are the total inborn and outborn (place of birth data). However, for analysis involving place of birth and outcomes, a few were missing either and thus 9,021 as stated in text – this is well captured in the methods and results.*

TABLE 4

 All the variables should be logically ordered by their values to facilitate reading.

*This has been actioned in the new version of this paper.*  

OVERALL

 In addition, the authors should address all the discrepancies and contradictions between the text and the tables.

*We have done a full pass reconciling every number quoted in text against its source table.*

CONCLUSIONS

 I hope that the authors find these suggestions helpful and improve the manuscript which should make strong contribution to neonatal health issues in Sub-Sharan Africa and help achieve the Sustainable Development Goal (SDG) of reducing neonatal mortality.

*Thank you for the opportunity to improve this paper through your kind review.*
